# Development of high-resolution melting (HRM) assay to differentiate the species of *Shigella* isolates from stool and food samples

**DOI:** 10.1038/s41598-021-04484-1

**Published:** 2022-01-10

**Authors:** Babak Pakbin, Afshin Akhondzadeh Basti, Ali Khanjari, Wolfram Manuel Brück, Leila Azimi, Abdollah Karimi

**Affiliations:** 1 Institute for Life Technologies, University of Applied Sciences Western Switzerland Valais-Wallis, 1950 Sion 2, Sierre, Switzerland; 2 Pediatric Infections Research Center, Research Institute of Children’s Health, Shahid Beheshti University of Medical Sciences, Tehran, Iran; 3 Present Address: Department of Food Hygiene and Quality of Control, Faculty of Veterinary Medicine, University of Tehran, P.O. Box: 14155-6453, Tehran, Iran

**Keywords:** Microbiology, Molecular biology, Gastroenterology

## Abstract

*Shigella* species, a group of intracellular foodborne pathogens, are the main causes of bacillary dysentery and shigellosis in humans worldwide. It is essential to determine the species of *Shigella* in outbreaks and food safety surveillance systems. The available immunological and molecular methods for identifying *Shigella* species are relatively complicated, expensive and time-consuming. High resolution melting (HRM) assay is a rapid, cost-effective, and easy to perform PCR-based method that has recently been used for the differentiation of bacterial species. In this study, we designed and developed a PCR-HRM assay targeting *rrsA* gene to distinguish four species of 49 *Shigella* isolates from clinical and food samples and evaluated the sensitivity and specificity of the assay. The assay demonstrated a good analytical sensitivity with 0.01–0.1 ng of input DNA template and an analytical specificity of 100% to differentiate the *Shigella* species. The PCR-HRM assay also was able to identify the species of all 49 Shigella isolates from clinical and food samples correctly. Consequently, this rapid and user-friendly method demonstrated good sensitivity and specificity to differentiate species of the *Shigella* isolates from naturally contaminated samples and has the potential to be implemented in public health and food safety surveillance systems.

## Introduction

*Shigella* is the main cause of bacillary dysentery or shigellosis. It is an endemic infectious intestinal disease throughout the world and one of the major causes of mortality and morbidity, mostly among children up to 5 years old, especially in developing countries^1^. The main symptoms of bacillary dysentery include severe bloody diarrhoea accompanied by gastrointestinal cramps. However, the general symptoms of *Shigella* intestinal infections range from mild watery to acute diarrhoea depending on the type of the species which causes the infection^2^. Bacillary dysentery is caused by one of the species of *Shigella* consisting of *S. dysenteriae, S. flexneri, S. Boydii,* and *S. sonnei*. The infectious dose of *Shigella* species is very low from 10 (for *S. dysenteriae*) to 100 bacterial cells (for *S. sonnei*). Because of their low infectious dose, the control of outbreaks caused by *Shigella* species is difficult^3^. In addition, due to the release of Shiga-toxin, *S. dysenteriae* causes an acute extraintestinal disease in humans, hemorrhagic uremic syndrome (HUS)^4^. Annually, more than 165 million cases and 1.1 million related deaths caused by *Shigella* infections are recorded around the world especially in developing and low-income countries. *S. sonnei* and *S. flexneri* are the predominant species of *Shigella* in developed and developing countries, respectively^5^. Foods are the main serious route of *Shigella* species transmission to humans, causing related foodborne intestinal and extraintestinal diseases^6^. Reliable, rapid, and accurate differentiation between the species of *Shigella* is crucial to evaluate the suspected food and clinical samples in public health and food safety surveillance systems^7^.

*Shigella* strains are susceptible. While they are excreted in a sufficient number in food and stool, they die off rapidly due to environmental conditions, including pH and temperature. Therefore, classic microbiological methods used to identify and differentiate *Shigella* species isolated from food and stool samples are relatively time-consuming, expensive and inefficient^8^. Because of a low infectious dose, low numbers of causative bacteria in food and clinical samples, inappropriate sampling and competition from other commensal bacteria, the diagnosis of *Shigella* species still remains obscure^9^. To date, few methods have been designed and developed for the diagnosis of shigellosis and the identification of *Shigella* species in industrial and developing countries. The standard gold method to discriminate *Shigella* species are species-specific serologic assays^10^. Also, molecular methods have been developed to differentiate *Shigella* species such as conventional multiplex PCR^8^, immunocapture PCR^11^, matrix-assisted laser desorption ionisation-time of flight mass spectrometry (MALDI-TOF MS)^12^, microarrays^13^, liquid chromatography-mass spectrometry (LC–MS)^14^ and next-generation sequencing (NGS)^10^ techniques. These assays have the advantage of being rapid. However, they are also expensive and complicated in their implementation. We conclude that a simple, rapid, inexpensive, accurate, specific, and sensitive method must be designed and developed for differentiation of *Shigella* species isolated from stool and food samples^10,15,16^.

High resolution melting (HRM) is an assay which, coupled with PCR, is regarded as a simple, low cost, and rapid method to detect single nucleotide polymorphism (SNP)^17^. Based on the dissociation behaviour of the amplicons, the HRM assay characterises and discriminates the PCR products in a single reaction tube without any additional instruments or protocols. The method is easy to perform, and the results can be obtained within approximately two hours^18^. Because the main inherent drawback of the HRM assay is its inability to identify the different species of a strain in a single reaction tube simultaneously, several researchers strongly recommended it to be developed and used in diagnostic systems^19^. PCR-HRM assay has already been used successfully to differentiate species of bacterial pathogens such as *Staphylococcus*^20^*, Listeria*^21^*, Cronobacter*^22^*, Salmonella*^23^*, Mycobacteria*^24–26^*, Pasteurella*^27^*, Campylobacter*^28^, *Yersinia*^29^, and *Brucella*^30^ isolated from food and clinical samples. Thus far, no studies have focused on designing and developing PCR-HRM assays to distinguish *Shigella* species from each other. In this study, we developed a PCR-HRM method to differentiate four species of *Shigella* isolated from stool and food samples.

## Results

### Design of the primers

**Figure 1:**
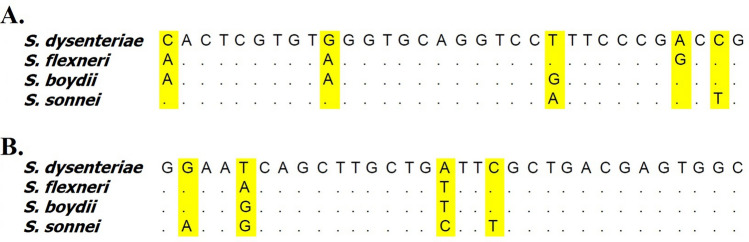
Nucleotide sequence alignments of *S. dysenteriae* (GenBank accession NC_007606), *S. flexneri* (NC_004741), *S. boydii* (NC_010658) and *S. sonnei* (NC_007384). (**A**) Primer pair purA-F-A and (**B**) primer pair rrsA-F-R.

Using the specific sequences reported for adenylosuccinate synthetase and 16 s rRNA encoding genes of *Shigella* species, two pairs of primers, including purA-F-R and rrsA-F-R (Table 1), were designed and evaluated in this study. PurA-F-R and rrsA-F-R primers were designed to target the SNPs in adenylosuccinate synthetase and 16 s rRNA encoding genes, respectively unique to each species of *Shigella* to differentiate them from each other accurately. Primers purA-F-R and rrsA-F-R amplified 83 and 92 bp fragments, respectively (Table 1). The theoretical or *in-silico* melting temperatures calculated by uMelt Quartz online tool for *S. dysenteriae*, *S. flexneri*, *S. Boydii,* and *S. sonnei* with purA-F-R primers were 70.6, 70.4, 70.1 and 69.5 °C, respectively. While for rrsA-F-R primers melting temperatures were 71.6, 71.9, 72.6 and 72.1 °C for *S. dysenteriae*, *S. flexneri*, *S. boydii,* and *S. sonnei*, respectively. As shown in Fig. 1, at least 2 different bases in the amplified sequences for both purA-F-R and rrsA-F-R primers in each *Shigella* species resulted in significant differences in the HRM profiles and melting temperatures. Both primers were also be able to amplify specific regions of *Shigella* species DNA by conventional PCR method. The purA-F-R primers were be able to generate the amplicons from 55 to 58.5 °C. However, rrsA-F-R primers amplicons were generated from 57.5 to 60 °C. In this study, we found that the optimal annealing temperatures for purA-F-R and rrsA-F-R primers in PCR-HRM assay were 58 and 59 °C, respectively. HRM investigated the effect of variations in these amplified sequences allowed us to differentiate *Shigella* species isolated from food and clinical samples.

**Table 1:** Primer sequences used to identify and differentiate *Shigella* spp. by HRMA.

Gene target	Primer sequence (5′-3′)	Amplicon size (bp)	Reference
*purA*	F-CTGGGTATCCTCAAAGCTTACTC	83	This study
	R-AGGAACTCGCCAGTTTCATC		
*rrsA*	F-ATGCAAGTCGAACGGTAACA	92	This study
	R-CCCTCCATCAGGCAGTTTC

### HRM with the reference strains

**Figure 2:**
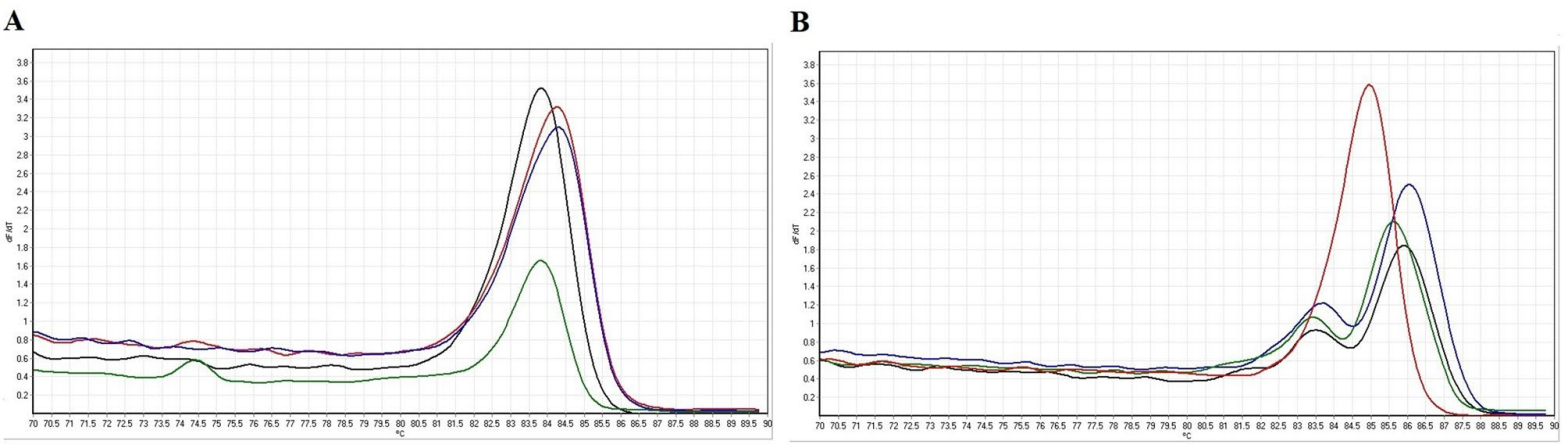
Melting curves of the reference strains of *Shigella* species analysed by PCR-HRM using (**A**) purA-F-R and (**B**) rrsA-F-R primer pairs. Green curve: *S. dysenteriae*. Red curve: *S. flexneri*. Blue curve: *S. boydii*. Black curve: *S. sonnei*.

**Figure 3:**
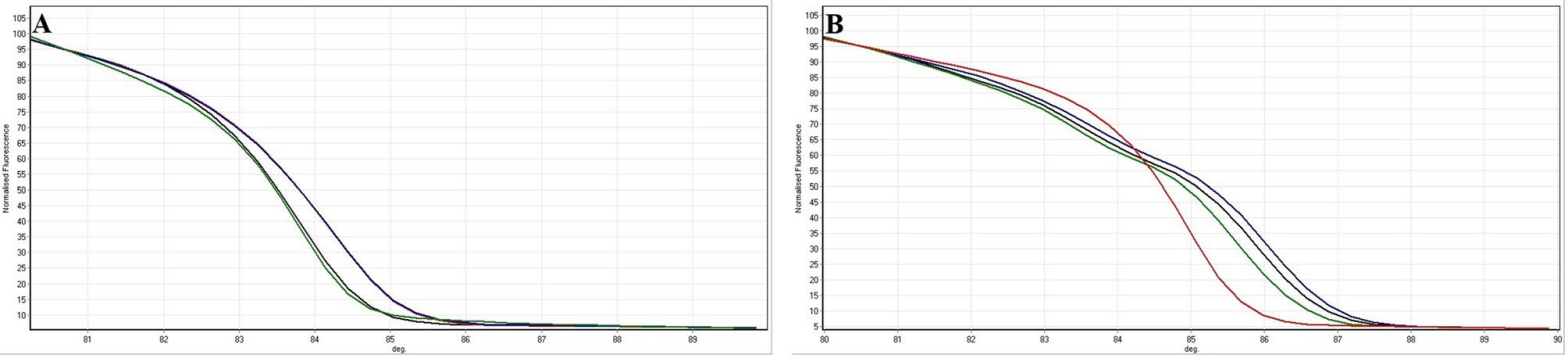
Normalised melting curves of the reference strains of *Shigella* species analysed by PCR-HRM using (**A**) purA-F-R and (**B**) rrsA-F-R primer pairs. Green curve: *S. dysenteriae*. Red curve: *S. flexneri*. Blue curve: *S. boydii*. Black curve: *S. sonnei*.

**Figure 4:**
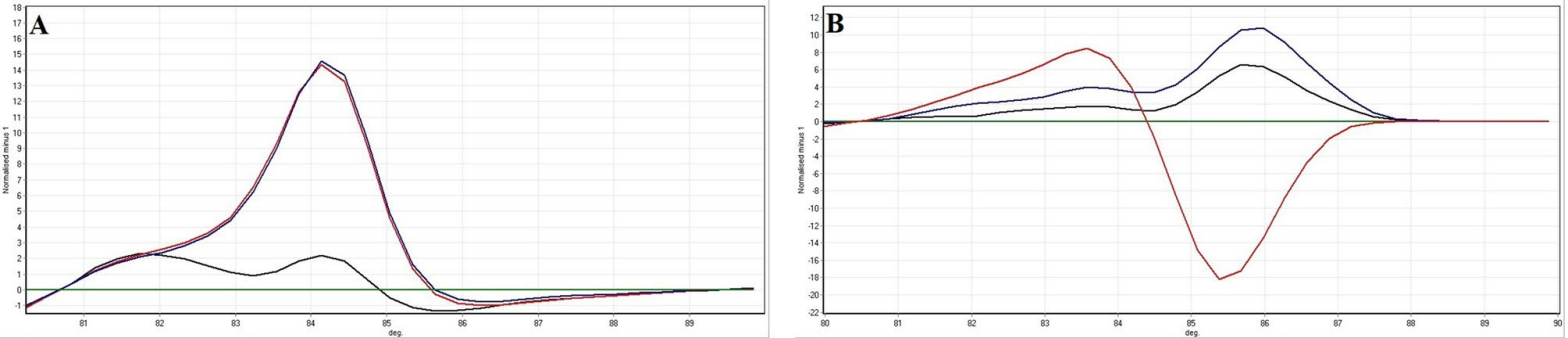
Difference plots of the reference strains of *Shigella* species analysed by PCR-HRM using (**A**) purA-F-R and (**B**) rrsA-F-R primer pairs. Green curve: *S. dysenteriae*. Red curve: *S. flexneri*. Blue curve: *S. boydii*. Black curve: *S. sonnei*.

Corresponding melting curves with definite melting temperatures were obtained from amplification of cultured reference strains tested in the PCR-HRM assay in this study. Figure 2A, B showed the HRM *Shigella* species-specific corresponding melting curves of the amplicons using purA-F-R and rrsA-F-R primers, respectively. Also, normalised curves and difference plots of the PCR-HRM assay using both primers (A: purA-F-R and B: rrsA-F-R) are demonstrated in Figs. 3 and 4, respectively. Melting temperatures of the amplicons generated by purA-F-R and rrsA-F-R primers to differentiate *Shigella* species by PCR-HRM method are presented in Table 2. As shown in Table 2, there were no significant (*P* < 0.01) differences between the melting temperature of *S. dysenteriae* and *S. sonnei* as well as and *S. flexneri* and *S. boydii* amplicons generated by purA-F-R primers demonstrating that these primers were not be able to differentiate *Shigella* species (confidence level > 90%). However, the PCR-HRM method with rrsA-F-R primers, designed and developed in this study, discriminated the four cultured species of *Shigella* from each other successfully and significantly (*P* < 0.01). Regarding Figs. 2, 3 and 4, normalised curves and difference plots also demonstrated that four species of *Shigella* could be differentiated by the PCR-HRM assay using rrsA-F-R primers (confidence level > 90%). However, purA-F-R primers were not able to distinguish *Shigella* species from each other correctly. Consequently, we were encouraged to use and evaluate PCR-HRM assay using rrsA-F-R primers, which have successfully been designed and developed at the present study and were be able to distinguish the *Shigella* species correctly for differentiation of *Shigella* species isolated from clinical and food samples (confidence level > 90%).

**Table 2:** Melting temperatures in PCR-HRM assay of the control strains of *Shigella* species.

*Shigella* spp.	T_m_ ± SD (°C)—Primer *purA*	T_m_ ± SD (°C)—Primer *rrsA*
*S. dysenteriae*	83.75 ± 0.05^a^	85.61 ± 0.04^a^
*S. flexneri*	84.28 ± 0.06^b^	84.69 ± 0.05^b^
*S. Boydii*	84.24 ± 0.05^b^	86.03 ± 0.05^c^
*S. sonnei*	83.85 ± 0.06^a^	85.90 ± 0.06^d^

*SD* Standard deviation, *T*_*m*_ Melting temperature.

Different letters in each column showed significant differences (*P* < 0.01).

### Sensitivity and specificity of the PCR-HRM method

**Figure 5:**
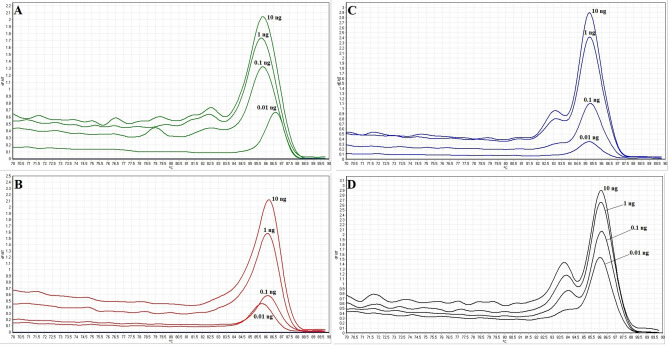
Melting curves of DNA tenfold dilution series for (**A**) *S. dysenteriae*, (**B**) *S. flexneri*, (**C**) *S. boydii* and (**D**) *S. sonnei*.

**Figure 6:**
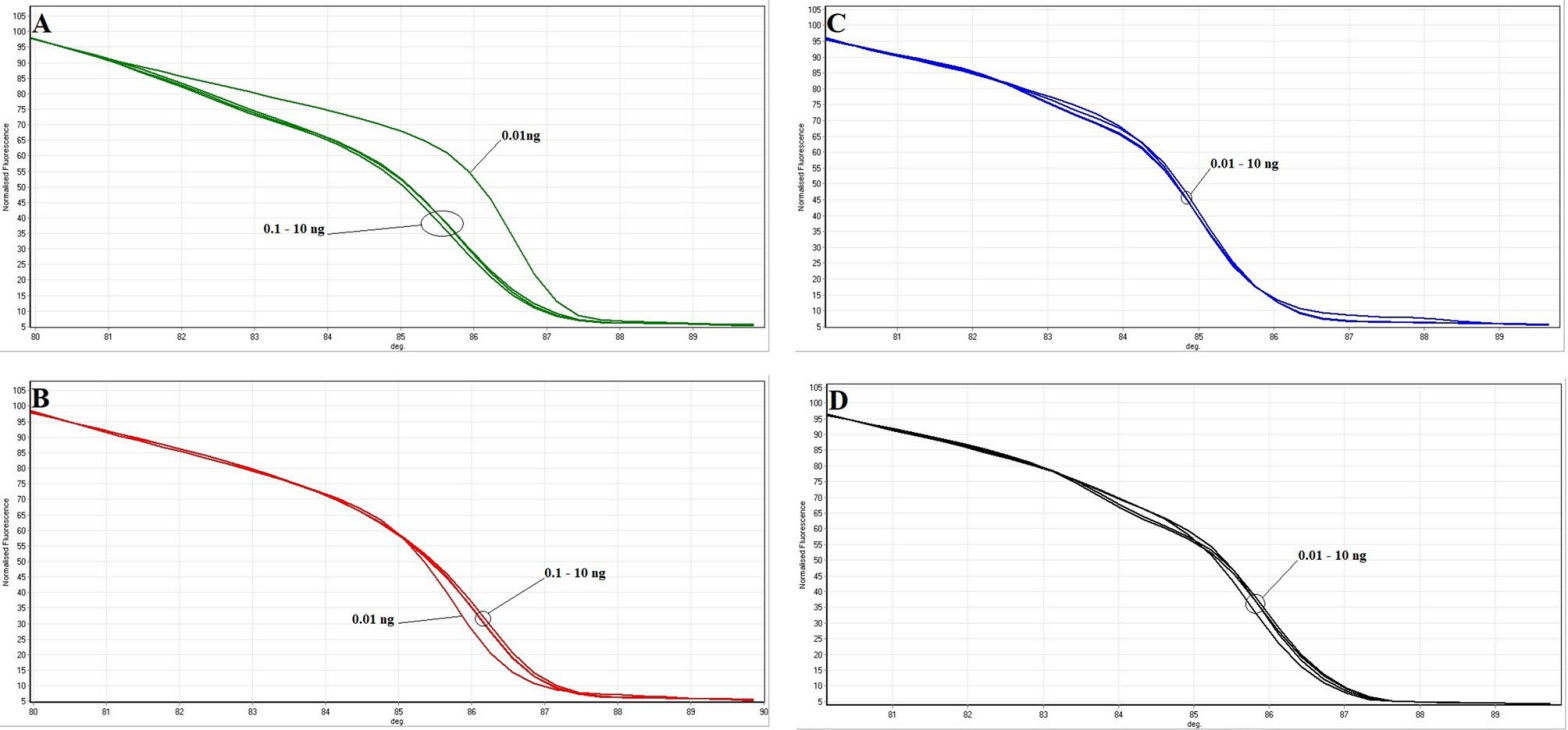
Normalised melting curves of DNA tenfold dilution series for (**A**) *S. dysenteriae*, (**B**) *S. flexneri*, (C) *S. boydii* and (D) *S. sonnei*.

In this study, we found that the PCR-HRM assay using rrsA-F-R primers was able to differentiate the Shigella species correctly. PurA-F-R primers did not. As a result, the specificity of the PCR-HRM assay was calculated 100% for the identification and differentiation of all *Shigella* species. The sensitivity or limit of detection of the assay was measured by analysis of the serial dilutions of the DNA templates using the PCR-HRM assay (confidence level > 90%). Melting and normalised melting curves of the serially diluted DNA templates analysed by PCR-HRM to identify four species of *Shigella* are shown in Figs. 5 and 6, respectively. The lowest concentrations of DNA by which the *S. dysenteriae*, *S. flexneri*, *S. Boydii,* and *S. sonnei* strains were identified correctly using the PCR-HRM assay, was 0.1, 0.1, 0.01 and 0.01 ng, respectively (confidence level > 90%). Consequently, the assay's sensitivity or limit of detection to identify S. boydii and S. sonnei was significantly (*P* < 0.01) more than that to identify S. dysenteriae and S. flexneri strains, which is indicated in Table 3. Identification of each species was compared with the reference strain in each reaction (confidence level > 90%).

**Table 3:** Melting temperatures (T_m_ ± SD (°C)) in PCR-HRM assay of melting curves of DNA tenfold dilution series for all *Shigella* species using rrsA primer.

*Shigella* spp.	DNA concentrations			
	10 ng	1 ng	0.1 ng	0.01 ng
*S. dysenteriae*	85.65 ± 0.03^a^	85.60 ± 0.03^a^	85.60 ± 0.02^a^	86.60 ± 0.04^b^
*S. flexneri*	86.30 ± 0.05^a^	86.32 ± 0.02^a^	86.30 ± 0.05^a^	85.70 ± 0.03^b^
*S. Boydii*^NS^	85.30 ± 0.01	85.32 ± 0.03	85.33 ± 0.03	85.30 ± 0.02
*S. sonnei*^NS^	86.10 ± 0.03	86.10 ± 0.01	86.15 ± 0.03	86.05 ± 0.03

*SD* Standard deviation, *T*_*m*_ Melting temperature, *NS* Not significant, Different letters in each row showed significant differences (*P* < 0.01).

### HRM with the isolates from clinical and food samples

**Figure 7:**
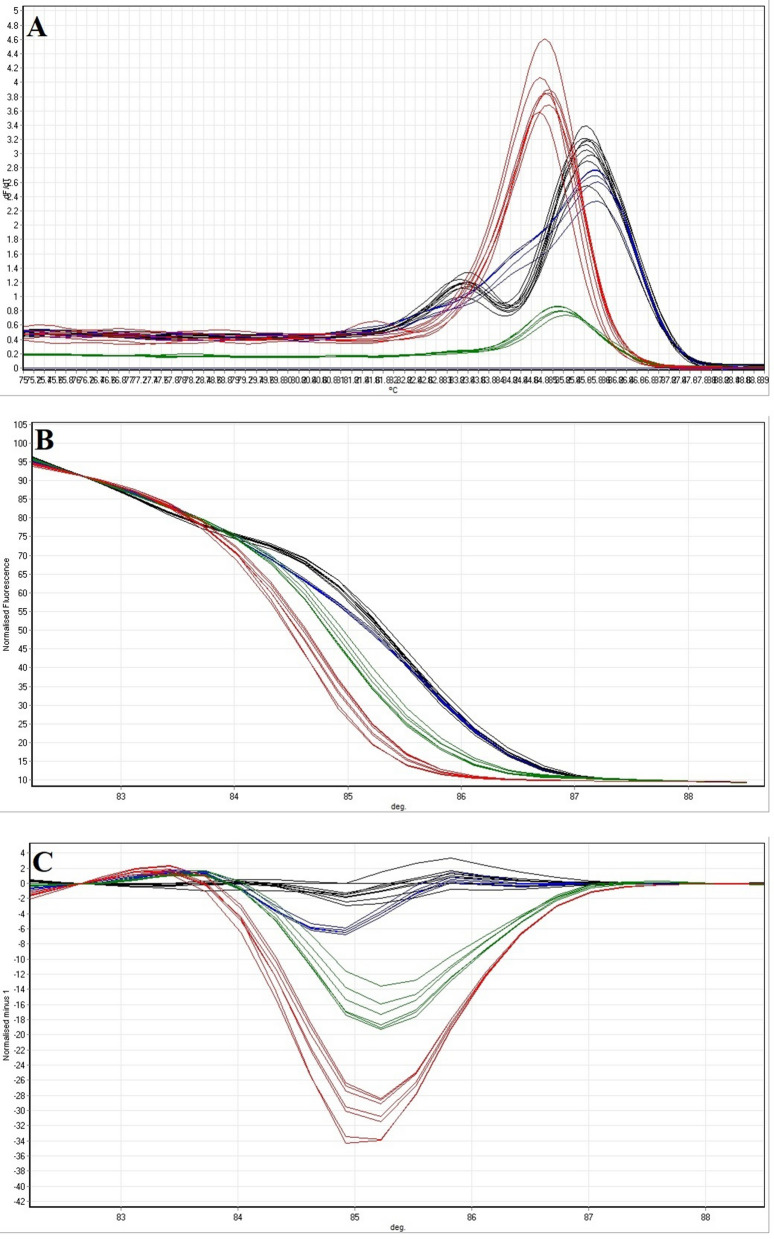
(**A**) Melting curves, (**B**) normalised melting curves and (**C**) difference plots the *Shigella* isolates from stool samples analysed by PCR-HRM assay. Green curve: *S. dysenteriae*. Red curve: *S. flexneri*. Blue curve: *S. boydii*. Black curve: *S. sonnei*.

**Figure 8:**
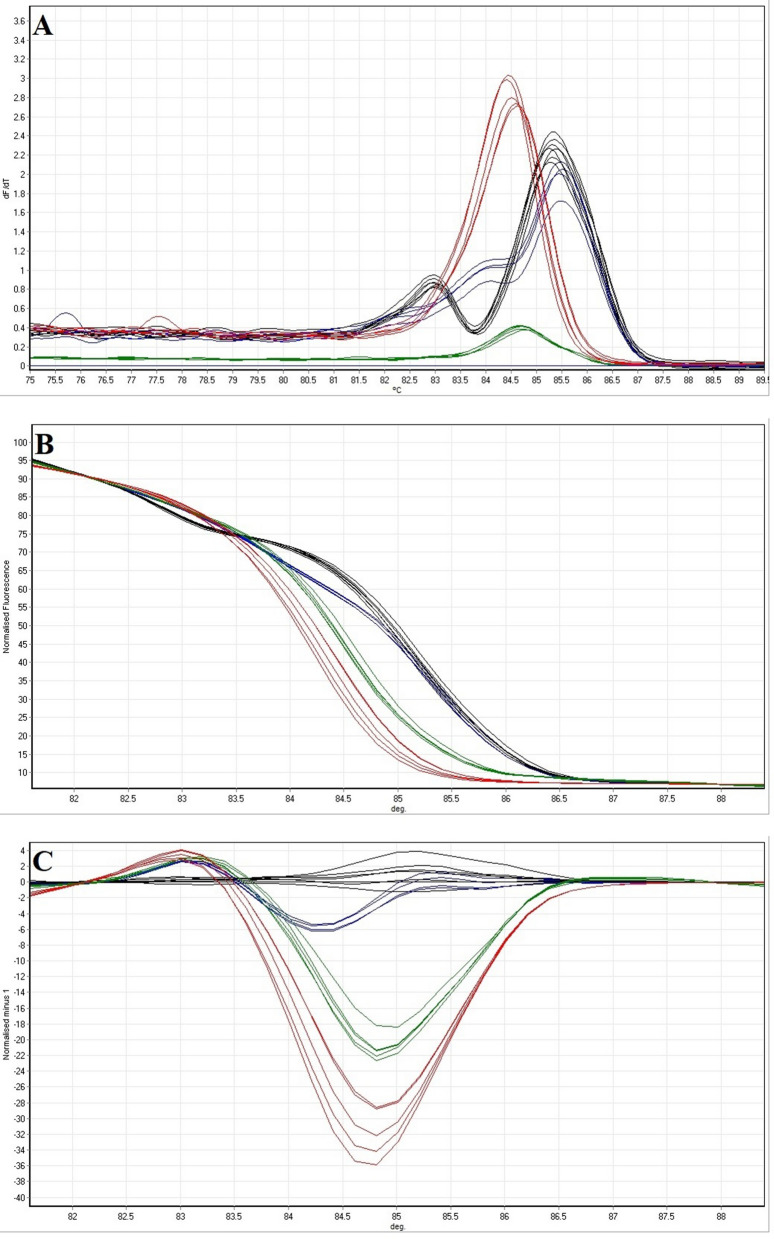
(**A**) Melting curves, (**B**) normalised melting curves and (**C**) difference plots the *Shigella* isolates from food samples analysed by PCR-HRM assay. Green curve: *S. dysenteriae*. Red curve: *S. flexneri*. Blue curve: *S. boydii*. Black curve: *S. sonnei*.

Out of 412 stool specimens from children up to 5 years old with acute diarrhoea and 470 food samples including raw milk, minced meat and vegetable salad samples, 28 and 21 *Shigella* species were isolated, respectively. All *Shigella* isolates (N = 49) were detected using the conventional culture-based method and identified by serological tests as the gold standards. *Shigella* isolates from clinical (6 *S. dyenteriae*, 7 *S. flexneri*, 5 *S. boydii* and 10 *S. sonnei* isolates) and food samples (5 *S. dyenteriae*, 5 *S. flexneri*, 4 *S. boydii* and 7 *S. sonnei* isolates) are presented in Table 4. Presumptive *Shigella* isolates from food and clinical samples were subjected to DNA extraction and then PCR-HRM analysis using the rrsA-F-R primers designed and developed successfully in this study. High resolution melting graphs including melting curve, normalised melting curve and difference plots corresponding to the PCR-HRM of the *Shigella* isolates from clinical and food samples, are demonstrated in Figs. 7 and 8, respectively.

**Table 4:** *Shigella* species isolated from clinical and food samples.

Bacterial species	Strain designation	Source	Serogroup
*S. dysenteriae*	MMRC2017241	Stool	A
*S. dysenteriae*	MMRC2017242	Stool	A
*S. dysenteriae*	MMRC2017243	Stool	A
*S. dysenteriae*	MMRC2017244	Stool	A
*S. dysenteriae*	MMRC2017245	Stool	A
*S. dysenteriae*	MMRC2017246	Stool	A
*S. dysenteriae*	PHSMS2019443	Food	A
*S. dysenteriae*	PHSMS2019444	Food	A
*S. dysenteriae*	PHSMS2019445	Food	A
*S. dysenteriae*	PHSMS2019446	Food	A
*S. dysenteriae*	PHSMS2019447	Food	A
*S. flexneri*	MMRC2017247	Stool	B
*S. flexneri*	MMRC2017248	Stool	B
*S. flexneri*	MMRC2017249	Stool	B
*S. flexneri*	MMRC2017250	Stool	B
*S. flexneri*	MMRC2017251	Stool	B
*S. flexneri*	MMRC2017252	Stool	B
*S. flexneri*	MMRC2017253	Stool	B
*S. flexneri*	PHSMS2019448	Food	B
*S. flexneri*	PHSMS2019449	Food	B
*S. flexneri*	PHSMS2019450	Food	B
*S. flexneri*	PHSMS2019451	Food	B
*S. flexneri*	PHSMS2019452	Food	B
*S. boydii*	MMRC2017254	Stool	C
*S. boydii*	MMRC2017255	Stool	C
*S. boydii*	MMRC2017256	Stool	C
*S. boydii*	MMRC2017257	Stool	C
*S. boydii*	MMRC2017258	Stool	C
*S. boydii*	PHSMS2019453	Food	C
*S. boydii*	PHSMS2019454	Food	C
*S. boydii*	PHSMS2019455	Food	C
*S. boydii*	PHSMS2019456	Food	C
*S. sonnei*	MMRC2017259	Stool	D
*S. sonnei*	MMRC2017260	Stool	D
*S. sonnei*	MMRC2017261	Stool	D
*S. sonnei*	MMRC2017262	Stool	D
*S. sonnei*	MMRC2017263	Stool	D
*S. sonnei*	MMRC2017264	Stool	D
*S. sonnei*	MMRC2017265	Stool	D
*S. sonnei*	MMRC2017266	Stool	D
*S. sonnei*	MMRC2017267	Stool	D
*S. sonnei*	MMRC2017268	Stool	D
*S. sonnei*	PHSMS2019457	Food	D
*S. sonnei*	PHSMS2019458	Food	D
*S. sonnei*	PHSMS2019459	Food	D
*S. sonnei*	PHSMS2019460	Food	D
*S. sonnei*	PHSMS2019461	Food	D
*S. sonnei*	PHSMS2019462	Food	D
*S. sonnei*	PHSMS2019463	Food	D

Regarding the melting curve, the normalised melting curve and difference plots, the PCR-HRM assay using rrsA-F-R primers was able tosignificantly categorise (*P* < 0.01) the amplicons into the four distinct main groups and showed a correct and successful *Shigella* species identification (confidence level > 90%). Consequently, the PCR-HRM method developed in this study succeeded in detecting and differentiating all four species of *Shigella* isolates from clinical and food samples. These results also confirmed the high specificity and efficiency of the assay to differentiate *Shigella* species in naturally contaminated samples demonstrating the potential application of this method to the analysis of the isolates from clinical and food samples.

## Discussion

Four species of *Shigella* including *S. dysenteriae*, *S. flexneri*, *S. Boydii,* and *S. sonnei* are among the main public health threats and food safety concerns around the world in developing and developed countries^3,5^. Nowadays, rapid and conventional methods such as hybridisation probes, multiplex PCR with species-specific primers, biochemical identification and serologic tests, which are so expensive or time consuming, have been developed and used to identify and differentiate four species of *Shigella* in food and clinical isolates^10,14,16^. Elahi et al. recently reported the development of a fluorescence DNA probe nano-biosensor method based on iron and gold nanoparticle to identify different species of *Shigella*^31^. However, this method is more expensive and complicated due to the need for four different probes bound to the gold and iron nanoparticles and the fluorescence spectrophotometer system to measure the fluorescence intensity and characterise possible interactions between the nanoparticles^32^. PCR-HRM is a rapid, simple, and cost-effective assay to detect SNPs in the PCR amplicons of the same size and has been highly recommended by several researchers to be developed and used for differentiation of pathogenic bacterial species isolated from clinical and food samples. It is worth noting that PCR-HRM assay can differentiate the species based on SNPs while the SNPs contribute to significant changes in the melting curves of the amplicons ^33,34^. This study developed a PCR-HRM assay to identify and differentiate four species of *Shigella* and used it as an efficient method to differentiate all *Shigella* species isolated from reference cultured, naturally contaminated clinical and food samples.

We designed and developed two primer pairs, including purA-F-R and rrsA-F-R to amplify specific sequences of adenylosuccinate synthetase and 16 s rRNA encoding genes in Shigella species and distinguish the species from each other based on SNP differences among the amplified sequences by PCR-HRM assay. In order to develop an HRM assay and effective primer design, the amplified PCR products should not exceed than 150 bp^35^. Both of these genes are conserved across the *Shigella* species and can be considered appropriate targets for PCR-HRM assay development to differentiate the species^36^. Ojha et al. developed a multiplex PCR assay and targeted *invC*, *rfc*, *wbgZ* and *rfpB* genes to discriminate the species of *Shigella*^8^. Radhika et al. also used *ipaH*, *wzy*, *wbgZ* and *invA* genes to design and develop a multiplex PCR method for differentiation of *Shigella* species^37^. So far, no PCR-HRM assay and the associated primers have been designed and developed to distinguish all species of *Shigella*. We demonstrated that *Shigella* species in cultured reference strains could be differentiated by the PCR-HRM assay using rrsA-F-R primers in a single round of reaction. However, the species were not discriminated from each other with purA-F-R primers. The DNA melting temperature and profile are strongly associated with the genetic variation and SNPs distribution patterns in the amplified sequences^38^. We demonstrated that nucleotide variations within the amplified sequences using rrsA-F-R primers were higher than that of purA-F-R primers. Also, higher distance differences were observed between the *in-silico* melting temperatures of the amplified sequences when the rrsA-F-R primers were used than purA-F-R primers.

Regarding the unique melting temperatures, normalised curves and difference plots, *S. dysenteriae*, *S. flexneri*, *S. Boydii,* and *S. sonnei* can be reliably and clearly distinguished from each other by the designed and developed PCR-HRM assay using the rrsA-F-R primers in this study. It is worthwhile to note that, considering the T_m_ alone is not sufficient to differentiate the bacterial species^39^ and after a specific transformation of the melting curves into the normalised curve and difference plots by Rotor-Gene 6000 software, species of *Shigella* can be clearly discriminated into four distinct groups. Other studies also assessed the normalised curves and difference plots of the cultured reference strains^40^. The specificity of the assay was measured 100%. The principal feature and advantage of our developed PCR-HRM method compared to the previous studies is the simple, straightforward and cheap PCR-based method to differentiate all *Shigella* species. Landolt et al. targeted the *gyrB* gene to develop a PCR-HRM assay for identification and differentiation of different species of *Mycobacterium,* including *M. tuberculosis*, *M. microti*, *M. bovis,* and *M. capra*. They successfully discriminated *Mycobacterium* species from each other with the analytical specificity of 100% by PCR-HRM assay and reported this method rapid, specific, low cost, and easy to perform in a single reaction tube^24^. Miller et al. used a PCR-HRM assay for identification and differentiation of six different reference species of *Pasteurellaceae* and exhibited six distinct melting profiles sufficiently distinguishable based on their T_m_ values. They found PCR-HRM method-specific (100%), rapid and cost-effective compared to the sequencing-based methods to identify and discriminate the *Pasteurellaceae* species^27^. To determine the sensitivity or LOD of the primers, a tenfold dilution series of the input DNA templates were prepared and analysed by the PCR-HRM assay^23,40^. Our developed assay demonstrated a good analytical sensitivity with a LOD of 0.01–0.1 ng of the input DNA template to identify and differentiate the *Shigella* species when we used 30-cycle amplification PCR program. The melt temperature of the amplicons significantly shifted to a higher melting temperature when the concentration of the input DNA template decreased.

Consequently, the PCR-HRM assay is highly sensitive to the concentration of the DNA template^41^. Bender et al. evaluated PCR-HRM assay for identification of different pathogens, and they reported a suitable sensitivity with 0.5–1 ng of input DNA template when performing 29-cycle PCR amplification. When the PCR cycles is increased, the sensitivity of the assay is consequently improved. However, this contributed to non-specific amplification^42^.

It is essential to evaluate the molecular technique through analysis of the naturally contaminated samples^10,25–28,42^. We identified and differentiated the species of totally 49 *Shigella* isolates from clinical and food samples by the PCR-HRM assay using rrsA-F-R primers to evaluate this method for analysing samples collected from patients and foods. All 49 *Shigella* isolates were identified and distinguished correctly. We found this assay completely specific (100%) and sensitive (100%) to differentiate *Shigella* species isolated from stool and food samples showing concordant results with the previous studies. Landolt et al. evaluated the performance of PCR-HRM assay to identify different species of Mycobacterium isolates from 25 clinical specimens, and they identified 23 isolates (92%) correctly^24^. Slany et al.^20^, Souza et al.^29^, Hoseinpour et al.^28^, Wu et al.^43^ and Cai et al.^22^ identified different species of *Staphylococcus, Yersinia, Campylobacter, Helicobacter,* and *Cronobacter* strains, respectively using PCR-HRM assay. All of these studies identified the microbial species isolated from naturally contaminated samples with a desirable specificity and sensitivity^20,22,28,29,43^. However, one of the main limitations of PCR-HRM assay generally is its inability to simultaneously identify more than one target or species in a test tube^38^. To the best of our knowledge, we developed the first *Shigella* species differentiation based on PCR-HRM assay and demonstrated this assay specific and sensitive to identify the species of *Shigella* isolates from real samples. This novel simple, rapid, cost effective and efficient molecular technique is highly recommended for designing and development of different diagnostic and identification tests. However, one of the most drawback of HRM assays is disability identify more than one species simultaneously while a single pair of primer is used. The design and development of multiple PCR-HRM assays to differentiate *Shigella* species are suggested to be implemented for future investigations.

## Conclusions

We developed a new PCR-HRM assay to identify and differentiate four species of *Shigella* isolated from naturally contaminated clinical and food samples and presented the results. We designed and developed the PCR-HRM assay with the analytical specificity of 100% and good analytical sensitivity of 0.01–0.1 ng of input DNA template concentration, indicating that this rapid method is sufficiently sensitive and specific for analysis of the isolates from real samples. Our assay also differentiated the species of all 49 Shigella isolates from clinical and food samples successfully. It is expected that a user friendly, rapid, cost-effective, sensitive, specific, and accurate PCR-HRM assay could be developed and implemented in clinical and food microbiology laboratories to differentiate the species of *Shigella* infection and contaminations contributing to improving public health and food safety surveillance strategies around the world.

## Methods

### Samples and bacterial references

The clinical samples (N = 412, including stool specimens from children up to 5 years old with acute diarrhea referred to the pediatric emergency service and central lab of Qazvin children hospital, Qazvin, Iran) and the food samples (N = 470, including raw milk (n = 130), minced meat (n = 160) and vegetable salad (n = 180) samples from different local markets located in different areas of municipality of Qazvin, Iran) were collected during July 2017 to February 2020. All samples were transported in cool boxes containing ice blocks (4 °C ± 0.5) immediately to the central research laboratory, College of Veterinary Medicine, University of Tehran, Tehran, Iran, for further microbiological investigations. Four standard *Shigella* species including *S. dysenteriae* ATCC 13,313, *S. flexneri* PTCC 1865, *S. boydii* ATCC 12,030, and *S. sonnei* PTCC 1777 were used in this study as positive controls and reference strains. All bacterial cultures were purchased and obtained in lyophilised form from Pasteur Institute (Pasteur In., Tehran, Iran), inoculated in Trypticase Soy Broth (TSB, Merck, Germany) incubated at 37 °C overnight before use. All bacterial strains were subjected to DNA extraction.

### Detection and identification of *Shigella* species by culture-based methods in clinical and food samples

*Shigella* species were isolated and identified according to the methods described by Phiri et al.^44^ and Mokhtari et al.^45^. Using sterilised disposable inoculation loops, stool samples were directly plated and inoculated on xylose lysine deoxycholate (XLD) agar (Merck, Germany) and incubated for 24 h at 37 °C aerobically. Suspected colonies, including red ones on XLD agar were selected, isolated and subjected to biochemical tests. We used the method previously described by Ahmed and Shimamoto^46^ to detect and identify *Shigella* species in different food samples. 10 g or mL of each sample (minced meat, raw milk and vegetable salad) was mixed vigorously with 100 mL *Shigella* broth (Merck, Germany) supplemented with Novobiocin antibiotic (2 mg L^−1^) (Merck, Germany), homogenised at 260 rpm for 5 min and incubated anaerobically at 42 °C overnight. 100 µL of the enriched samples were streaked onto XLD agar (Merck, Germany) plate and incubated aerobically at 37 °C for 24 h. Suspected colourless or red colonies on XLD agar isolated from stool and food samples were subjected to the biochemical tests, including IMViC, TSI, motility, oxidase and urease production. Also, presumptive *Shigella* isolates were identified and grouped serologically using commercial *Shigella* genus and species antisera kits (Difco Co., MI, USA) to confirm the genus and determine the species of the *Shigella* isolates, respectively.

### DNA extraction

Presumptive *Shigella* isolates, colourless or red colonies on XLD agar, from food and clinical samples and the enriched reference bacterial strains were subjected to DNA extraction. Genomic DNA of the bacterial isolates and strains were extracted using the gram-negative bacterial DNA extraction kit (Sinaclon Co., Tehran, Iran) according to the manufacturer’s protocol. The quality and quantity of the extracted genomes were evaluated using a NanoDrop 2000 spectrophotometer instrument (ThermoFisher, MD, USA). The final concentrations of all extracted genomes were adjusted to 50 ng µL^−1^ and all templates were kept at − 20 °C until further analysis.

### Primer design

In this study, to differentiate *Shigella* species, the primers were designed based on targeting the highly conserved and variable regions of adenylosuccinate synthetase (*pur*) and 16 s rRNA (*rrs*) genes. The primers were designed according to the alignment of the available sequences of *purA* and *rrsA* genes in *Shigella* species. We used GeneBank (NCBI, USA) sequence accession numbers NC_007606.1, NC_004741.1, NC_010658.1 and NC_007384.1 for *S. dysenteriae*, *S. flexneri*, *S. boydii* and *S. sonnei*, respectively. The sequences were aligned using CLUSTALW (EBI; http://www.ebi.ac.uk/CLUSTALW). PrimerQuest IDT online software (Integrated DNA Technologies, Inc, San Diego, CA, USA; https://www.idtdna.com/pages/tools/primerquest) was used to design two pairs of primers, purA-F-R and rrsA-F-R, to differentiate four species of *Shigella*. The quality of the designed primers was assessed by OligoAnalyzer online tool version 3.1 (https://eu.idtdna.com/pages/tools/oligoanalyzer), and the primer specificity was evaluated using Primer-BLAST online tool (https://www.ncbi.nlm.nih.gov/tools/primer-blast/). The primers were synthesised and purchased from CinnaGen company (Tehran, Iran).

### Annealing temperature optimisation for primers

A temperature gradient program was designed and created in a thermocycler PCR machine (ABI, Applied Biosystems, CA, USA) to ensure that the primers designed and developed in this study were capable of amplification of the target regions in four *Shigella* species without any non-specific amplification and primer dimers demonstrating subsequent appropriate and desirable results in the following PCR-HRM analysis. Separate PCR reactions were performed for each pair of primers independently. PCR conditions included: 12.5 µL of 2X PCR master mix (Ampliqon, Denmark), 1 µL of each primer (20 µmol µL^−1^), 2 µL of DNA template (50 ng µL^−1^) and sterilised DNase-free water up to the final reaction volume. Thermocycling conditions were the initial denaturation step including one cycle of 94 °C for 5 min; followed by 30 cycles of denaturation at 94 °C for 30 s, annealing from 50 to 60 °C for 30 s and elongation at 72 °C for 20 s; and a final extension step at 72 °C for 5 min. The PCR products were characterised using gel electrophoresis on 2% w/v agarose/TBE buffer at 100 V for 60 min and visualised by UV transilluminator and gel documentation system (GelDoc model ccd-5, GenIranLab, Tehran, Iran). The primers were evaluated by using the reference strains and some Gram-positive and Gram-negative bacteria as the non-target strains.

### In-silico melting simulation

*In-silico* simulations of high-resolution melting curves were based on the target sequence regions amplified by purA-F-R (amplicon size: 83 bp) and rrsA-F-R (amplicon size: 92 bp) primers. The target sequences' theoretical melting temperatures (Tm) were calculated using the uMelt Quartz online tool version 3.6.2 (https://www.dna-utah.org/umelt/quartz/um.php).

### PCR-HRM

PCR-HRM was performed with a Solis-Bio 5X Evagreen® PCR-HRM hot start Master Mix (Solis-Bio Dyne, Tartu, Estonia) on a Rotor-Gene Q 6000 real-time PCR instrument (Corbett, Australia). PCR-HRM reaction tubes, performed for each primer pair separately, contained 4 µL of the 5X PCR-HRM master mix, 1 µL of each primer (10 µM), 2 µL of the DNA template (50 ng µL^−1^) and DNase-free water to a final reaction volume of 20 µL. The amplification procedure was carried out using the following conditions: an initial denaturation step at 94 °C for 12 min, followed by 30 cycles of denaturation at 94 °C 30 s and annealing-extension at 58 and 59 °C for purA-f-R and rrsA-F-R, respectively for 30 s; followed by HRM procedure with increasing temperature from 65 to 95 °C with data acquisition every 0.2 °C for both primer pairs. The sizes of amplicons produced by purA and rrsA primers were 83 and 92 bp, respectively (Table 1). The analysis of HRM was conducted using the Rotor-Gene 6000 software version 2.02 (Corbett, Australia) to determine the T_m_, melting and normalised melting curves for each species of *Shigella*. For each experiment, *S. dysenteriae* ATCC 13,313, *S. flexneri* PTCC 1865, *S. boydii* ATCC 12,030, and *S. sonnei* PTCC 1777 were included as the positive controls and melting curve standards.

### Sensitivity and specificity of the PCR-HRM method

To determine the sensitivity of the PCR-HRM method designed and developed in this study, serial tenfold dilutions (10, 1, 0.1 and 0.01 ng µL) of the DNA templates of each reference *Shigella* species separately were prepared and analysed by the PCR-HRM assay. The sensitivity of the assay was measured as the lowest concentration with successful PCR amplification at which the *Shigella* species can be identified correctly by the developed PCR-HRM method. To check the possible incorrect signals and the specificity of the assay, differentiation and identification of four species of *Shigella* were performed among the *Shigella* species isolated from clinical and food samples by both the conventional serology-based (the gold standard) and the developed PCR-HRM methods.

### Statistical analysis

One-way analysis of variance (ANOVA) was conducted to determine the significant (*P* < 0.01) differences among the groups of melting temperatures using SPSS version 23.0.0 (SPSS Inc., Chicago, IL, USA). Also, all experiments and measurements were performed in triplicates.

### Ethics approval

The sampling and study protocols were investigated and approved by the Ethics Committee of the College of Veterinary Medicine, University of Tehran (IR.UT.REC.1397.204). In this study, all research was performed in accordance with relevant guidelines/regulations and the Declaration of Helsinki. For all cases, informed consent was obtained from the parents of the patients whose stool specimen was included in this study.

## Data Availability

All raw data in this study are available from the corresponding author on a reasonable request.
